# Shifting seas: understanding deep-time human impacts on marine ecosystems

**DOI:** 10.1098/rstb.2024.0026

**Published:** 2025-07-10

**Authors:** Luke E. Holman, Kristine Bohmann, Oliver E. Craig, David Orton, Mikkel Winther Pedersen, Morten Tange Olsen, Ruth H. Thurstan, James Scourse

**Affiliations:** ^1^ Section for Molecular Ecology and Evolution, Globe Institute, University of Copenhagen, Copenhagen, Denmark; ^2^ BioArCh, Department of Archaeology, University of York, York, UK; ^3^ Centre for Ancient Environmental Genomics, Globe Institute, University of Copenhagen, Copenhagen, Denmark; ^4^ Centre for Ecology and Conservation, University of Exeter - Penryn Campus, Penryn, UK; ^5^ Department of Earth and Environmental Sciences, University of Exeter - Penryn Campus, Penryn, UK

**Keywords:** anthropogenic marine impacts, human–ocean interactions, marine conservation baselines, climate change and oceans, interdisciplinary environmental science, historical marine ecology, eDNA, marine biodiversity change, stable-isotope analysis, archaeozoology

## Abstract

Humans have interacted with, and impacted, marine ecosystems for millennia. During this time, the oceans have experienced ecosystem degradation through harvesting, habitat change, pollution, the introduction of invasive species and climate change. Despite extensive documentation of substantial recent anthropogenic impacts, our understanding of ancient marine biodiversity remains limited for many ocean regions. This theme issue advances our knowledge of past oceans, revealing how diverse perspectives from across disciplines can provide new insights into marine anthropogenic exploitation over thousands of years. Through engaging a range of source materials, including material remains, historical records and palaeoenvironmental archives, the contributions reveal shifting seas responding to both natural climatic changes and human impacts. Collectively, these outputs demonstrate the value of interdisciplinarity and cross-cultural approaches in understanding ocean change. As marine restoration programmes expand globally, combining disciplinary approaches and novel methods across deep time can provide novel baseline data against which to measure recovery and raise ambition for marine conservation. Beyond biodiversity baselines, understanding past ocean changes can provide key insights into the mechanisms through which human impacts alter marine ecosystems, allowing us to learn from our ancestors’ effective and ineffective ocean stewardship practices. Just as diverse ecosystems are buffered against change, diverse human–ocean interactions are important for flourishing future seas.

This article is part of the theme issue ‘Shifting seas: understanding deep-time human impacts on marine ecosystems’.

## 1. Troubled seas

Our ancestors encountered coastlines rich with marine life as they dispersed across the Earth hundreds of thousands of years ago. In their wake, early peoples left evidence of their interactions with the ocean, teaching us about the nature of this relationship. Cave art depicting marine creatures [1], marine mollusc shells in archaeological refuse sites (or middens) [2], ancient fish hooks [3], isotope analyses of human remains [4] and butchery marks on marine fish and mammal bone all show that marine foods were exploited by hominin ancestors from at least the Middle Palaeolithic [5–7], collectively demonstrating the long-term importance of the seas to the human story. Humans ventured to shallow water islands and along coastal areas, bringing them into contact with untouched coral reefs, seagrass meadows and rich shelf seas. Some authors suggest that productive marine habitats, such as kelp forests and mangroves, provided people with food and shelter and facilitated their dispersal along coastlines [8]. Only much later, in the last few thousand years, have humans undertaken sustained and systematic voyages across the open oceans, pushing into wild and untamed seas far out from land [9]. Finally, in the last hundred years, people have probed the deepest oceans, finding unimaginably diverse and unique marine life flourishing in the inky dark.

The influences of the ocean on humanity are manifold and complex. While our impacts on the ocean are similarly diverse, one stressor emerges consistently across the globe—extractive harvest. Generalizing the impact of people on the oceans across ecosystems and human cultures is difficult, but early hunter-gatherer-fisher populations are understood to have had limited large-scale impacts on marine ecosystems compared tosubsequent human periods [10]. Indigenous oyster fisheries across Europe, North America and Australia show persistent harvests across many thousands of years, providing an example of continuous human exploitation [11–13]. Despite this remarkable evidence for sustained marine harvest, our early ancestors also impacted the marine systems they exploited. In oyster fisheries, we observe decreases in average shell size in archaeological remains, suggesting strong harvesting pressure [12,14]. Moreover, there is evidence that supports the local extirpation of grey seals from the Baltic Sea during the Mesolithic period by hunter-gatherer-fishers [15]. Despite exploitation over many millennia, marine megafaunal extinctions have only occurred in recent centuries [16,17]. This contrasts with patterns in terrestrial ecosystems, where megafauna experienced declines and extinctions across continents as early as the Late Pleistocene as a consequence of human exploitation in combination with environmental changes [18].

Just as human societies have different subsistence patterns across space and time, human impacts across ancient marine ecosystems have been diverse and varied [19]. As societies across the world developed farming practices, fishing and vessel technologies also improved, allowing people in the early agricultural societies to travel further at sea and exploit coastal fisheries catching larger species [20]. The impacts on these newly available marine resources were just as variable as the impacts of previous hunter-gatherer-fishers [10]. Overall there is limited evidence for a wide-scale transformation of marine exploitation at the advent of farming [10,21], and major effects were likely to be localized in their extent [22]. For example, there is evidence for a reduction of the estimated length of the Atlantic cod [23] and stability of the Pacific cod length [24] shown by cod bones from archaeological refuse sites spanning several millennia. Later, continued technological development and global human population growth drove greater fishing efforts and the establishment of new fisheries.

Early Roman Empire art and literature depicted large species from high up the marine food web, and archaeological evidence across the ancient Roman period documents coastal fishing of Mediterranean tuna with elaborate net systems [25,26]. Later, the Norse expanded across the North Atlantic in a quest for walrus ivory, causing serial depletion and local extinctions of walrus stocks from Europe to the High Arctic [27]. Over the past millennium, reconstructed catch records from European decked wooden vessels document annual fish catches reaching hundreds of thousands of tonnes [28]. During this period, substantial whaling in the Bay of Biscay resulted in declines in Basque right whale catches from the sixteenth century [29]. A notable example of exploitation can be found in sixteenth-century Newfoundland, where annual catches of Atlantic cod exceeded hundreds of thousands of tonnes [30], while in recent decades, the catch remains well below fifteen thousand tonnes yearly [31]. Collectively, the evidence indicates expanding technologically driven marine organism consumption and the beginnings of globalized extraction, with long-range trade networks supplying urbanized populations from non-local fisheries [28,32,33]. Against the rising tide of hooks and nets, many post-medieval marine ecosystems exhibited remarkable resilience, evidenced by written accounts describing the oceans as seemingly inexhaustible [34], and localized decreases in catches were offset by expansion to new fishing areas and novel fishing technologies [22,28]. European colonization from the fifteenth century onwards had a devastating effect on many Indigenous Peoples, with genocide, displacement and dispossession homogenizing marine human–environment interactions towards massive globalized extraction [10,35].

The eighteenth and nineteenth centuries marked the beginning of the industrial period across Europe. Fishing efforts increased as stocks declined, and fishers could no longer switch to less-impacted proximal fisheries [36,37]. This marked a dramatic decline in the population of many fished species globally [37,38]. The severity of these anthropogenic impacts became evident as some finfish [39] and oyster [40,41] stocks from across the globe collapsed entirely in the twentieth century. Whales and seals were subject to extensive industrialized hunting and systemic eradication during the nineteenth and twentieth centuries, with records revealing dramatic losses for many populations [42–44]. Some marine taxa were even driven to extinction, for example, the great auk (*Pinguinus impennis*), Steller’s sea cow (*Hydrodamalis gigas*) and the Caribbean monk seal (*Neomonachus tropicalis*) [45–47], all as a result of human exploitation. The decline of marine life has continued or, in some cases, accelerated, in modern fisheries, with a peak and subsequent decline in average global catches in the late twentieth century [48]. However, this global average hides a decrease in the trophic level of catches across the same period [49]. Some authors suggest that lower trophic-level species are targeted as larger predators become less available, so-called fishing down the food web [50]. Other evidence, however, suggests that the addition of low trophic-level species, alongside continued fishing pressure on high trophic-level species—fishing through the food web, drives the observed decrease in trophic level [51]. Both perspectives see increasing fishing pressure further down the food web as a global modern phenomenon.

While fishing and hunting of marine life has been a key anthropogenic marine stressor, it is only one of a suite of mechanisms through which humans have affected marine biodiversity. Jackson *et al.* [52] proposed a historical sequence of human disturbance, always starting with fishing and then variably followed by pollution, mechanical habitat destruction, the introduction of invasive species and finally climate changes depending on the period and location. One of the earliest examples of marine pollution comes from the detection of elevated lead in Mediterranean sediment core layers dated to the Bronze Age associated with smelting and refining [53], but there is limited evidence supporting lead pollution as a major impact during this time. The first evidence for substantial pollution and habitat destruction/change comes from the historical period [54]. For example, deforestation, the construction of water mills and the farming of domestic species affected rivers through sedimentation across the last millennium in Europe, which in turn affected populations of fish dependent on migration between fresh and saltwater as part of their life cycle [55]. Pollution and habitat alteration significantly increased during industrialization. During this time, three-dimensional habitat-forming marine taxa, such as oyster reefs and eelgrass beds, were further depleted through sedimentation and trawling, reducing habitat for associated taxa whose populations suffered as a result [10]. The introduction of marine invasive species has been documented across the industrial and historical periods [56]. However, there is limited evidence for substantial impacts on ocean biodiversity as a result of introductions before the modern period [57].

Human impacts on marine ecosystems in recent decades have been global and substantial in magnitude [10,58,59]. In addition to the peak in fishing pressure, invasive species introductions, habitat alterations and some forms of pollution have all increased or plateaued [16,56]. Alongside these stressors, human-driven climate change has emerged as a ubiquitous threat. The effects of climate change on ecosystems and biodiversity are increasingly common across marine ecosystems [60,61], and interactions between climate change and other stressors, for example, overfishing or eutrophication, are likely to further degrade ecosystems [58]. Fishing is currently the largest direct driver of modern marine biodiversity loss, followed by climate change, pollution, sea-use change (analogous to habitat alteration) and invasive species introductions [62]. As these factors impact the global ocean, biodiversity change is ubiquitous across scales, with the vast majority of time-series datasets showing change in the modern period [63].

**Figure 1.**
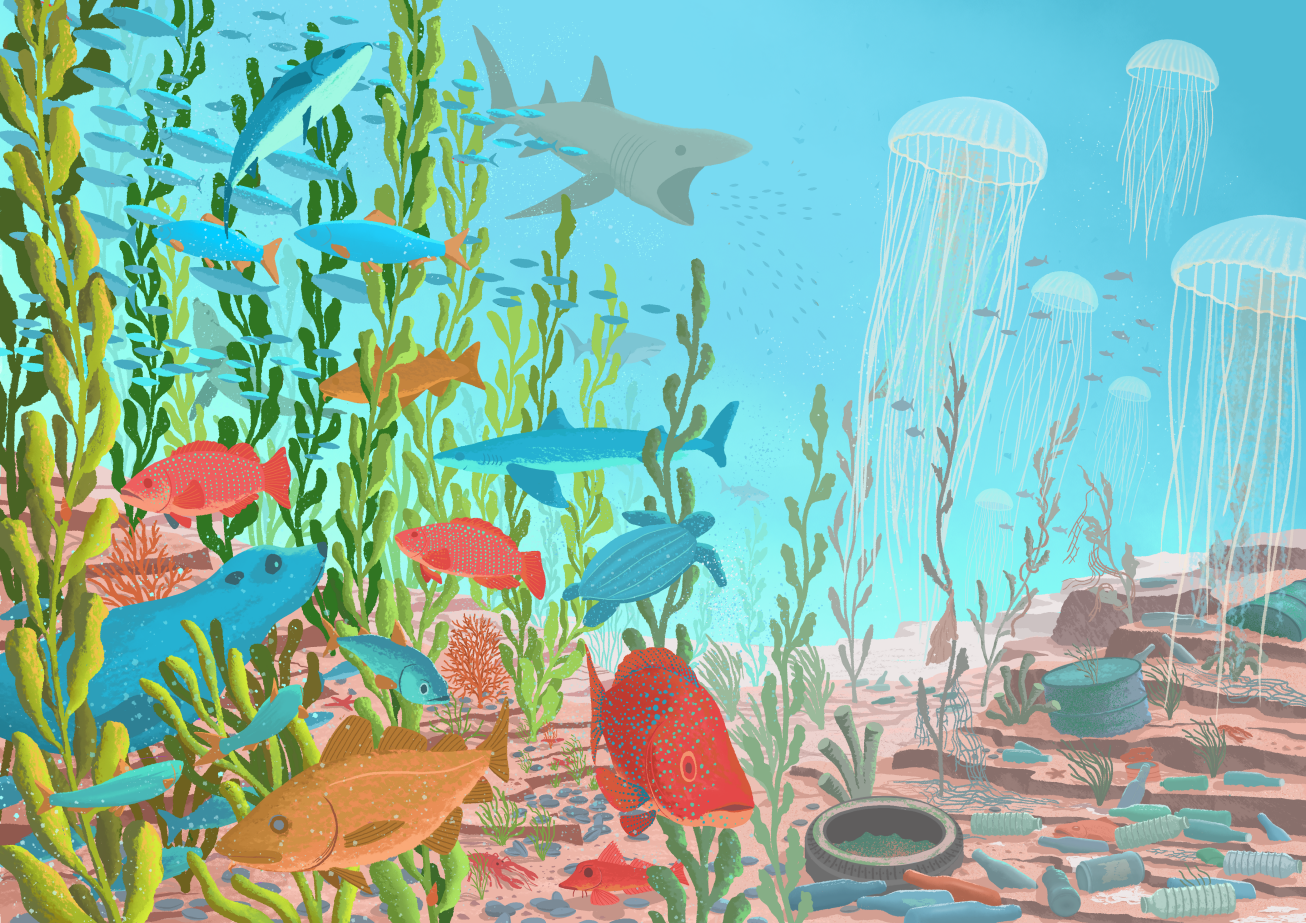
Humans have placed huge pressure on the oceans over recent decades. The diversity and abundance of ancient seas eclipse the degraded state of modern ecosystems. Depictions of ancient oceans, shown here on the left, can fill us with deep sadness and loss but also a sense of hope and purpose to transform and restore today’s degraded oceans, shown here on the right, back towards a flourishing healthy state. Illustration by Jacek Matysiak.

Never before have the seas faced as much pressure as they are today. However, our understanding of past marine biodiversity is equally unprecedented. This theme issue brings together articles that lift the fog of time across marine ecosystems and disciplinary divides, revealing more about the dramatic ways in which humans have impacted the seas and painting a picture of ancient oceans containing vast riches, with biodiversity and abundance incomparable to today’s degraded state (figure 1).

## 2. Diverse seas

From coral reefs to the deepest hydrothermal vents, marine ecosystems contain remarkable diversity. Similarly remarkable is the plethora of different approaches and disciplines used to understand and reconstruct the oceans. The earliest naturalists worked before the emergence of formal scientific fields and therefore often traversed very different domains of research, combining them in ways that became less common as a subject-specific focus became more prevalent. For example, Pliny the Elder’s *Naturalis Historia* [64] described zoological observations of creatures from the first century alongside anthropological descriptions of different peoples and cultures. More recently, Alexander von Humboldt’s accounts of South America combined a huge swathe of observational data to detail geological, atmospheric, botanical, zoological and limnological profiles of nature [65]. He found links between these natural history observations and other local observations that today would be siloed within the humanities—linguistics, art, culture [65,66].

Subsequent scientific progress saw increasing specialization and a narrowing of disciplinary focus. However, modern scientists are again incorporating growing interdisciplinarity in their work, revealing patterns and processes intractable to studies within a single field. For example, Human Ecodynamics is an approach emerging from archaeology that understands humans as a part of (and not separate from) the wider dynamic ecological system [67]. This view prompts the practitioner to see the social and natural world in a combined socio-ecological system changing through time, providing a wider and more complete understanding. Similar examples from different domains that reveal unique perspectives by broadening, rather than narrowing, the disciplinary aperture include One Health [68]; Planetary Health [69]; the Biomolecular Humanities [70]; and Sustainability Science [71].

While we are collectively beginning to appreciate the value of interdisciplinarity in finding novel conceptual ground, studies that inherently require interdisciplinary methods are also increasingly common. For example, the analysis of ancient DNA extracted from marine sediments requires expertise in geology for sampling, molecular biology for laboratory processing and sequencing, computer science for bioinformatics and community ecology for analyses. However, research that combines approaches or methods from more disparate disciplines to understand past oceans is more rare. Campana *et al.* [72] recently combined archaeological samples, fish ear bone (otolith) stable-isotope analysis and fisheries statistics to reveal human fishing impacting Icelandic cod populations as early as the fifteenth century—a finding that would elude single-discipline research. de Kock *et al.* [73] identified the ancient foraging grounds of Mediterranean green sea turtles by combining modern stable isotope and satellite tracking data with bioarchaeological techniques (palaeoproteomics and stable-isotope analysis). The results highlight the importance of a threatened ecosystem, North African seagrass meadows, as a critical sea turtle foraging ground across millennia. Crucially, while both these contributions combine disciplines within the broader framework of western science, it is important to outline that other ocean perspectives and knowledge systems exist and have a valuable contribution to understanding the seas.

As outlined in the first section, Indigenous Peoples have interacted with marine ecosystems for millennia, with the oceans providing sustenance [5], influencing art and expression [1] and forming a central part of the shared common memory of Indigenous cultures across time to the present day [74]. Moreover, researchers are now beginning to recognize that ecological functions can be provided by some place-based societies (*sensu* Salomon & McKechnie [75]—communities whose identities, knowledge and livelihoods are deeply rooted in long-term, reciprocal relationships with specific local ecosystems). This perspective considers people as a part of, rather than separate from, marine ecosystems as nutrient cyclers, ecosystem engineers, dispersers and other roles typically attributed to other marine organisms [75]. As with non-human ecosystem engineers, Indigenous ecosystem management can result in substantial shifts in local ecologies, but overall there is a strong contrast with the dominant post-colonization western relationship, which has until recently been characterized by pervasive unsustainable extraction [16]. Indigenous oyster fisheries again offer a useful perspective, with examples of extraction over millennia across present-day Europe, North America and Australia with minimal overall impact [11,12]. The mechanisms of management in these cases are likely diverse and contextual, but the outcomes prompt us to evaluate how we can promote the coexistence of Indigenous ocean perspectives alongside western science. Some possibilities from around the world are given by Reid *et al.* [76], and one notable example is ‘Two-Eyed Seeing’ (Etuaptmumk in Mi’kmaw). This framework uses the analogy of looking through two eyes, one with the strengths and perspectives of Indigenous knowledge and a second with mainstream knowledge and ways of seeing. Together the entire perspective is greater than the sum of the parts, changing the perceiver and prompting action. This framework holds great promise in helping us comprehend past oceans because a complete understanding requires not only interdisciplinary knowledge from our western science ‘eye’, but allows us to see more fully by incorporating Indigenous ecological knowledge and experience into our perception.

We have attempted in this issue to provide diverse perspectives, but many contributions come from western scientists working in the Global North, and our collective understanding of marine change in underrepresented regions is particularly sparse. We make no claim to be able to render the complex, manifold relationships with the ocean across the world into a single issue, nor do we believe that such a publication would be possible simply by incorporating contributions across wider geographic areas through time. Instead, the contributions presented here demonstrate the value of bringing together diverse perspectives, even if they represent a biased subset of the whole, to provide clearer knowledge on how the oceans have changed over time.

## 3. Shifting seas

This issue brings together examples of sea changes from across the globe, with contributions documenting minimally exploited marine biodiversity and heavily degraded ecosystems. All articles cover a substantial period of time in their approach and highlight how climate, culture and ecosystems are embedded in a dynamic nexus, with each affecting the others in unexpected and complex ways.

### (a) Marine biogenic archives

The first group of articles demonstrate what can be learnt from the bones and shells of animals. It is a common experience to find a shell while walking down a beach and wonder about the life of the animal that created it. These articles use a range of disciplines to extract data from animal remains, forming an archive that documents the scale and timing of marine ecosystem changes, thus enabling us to interpret the likely drivers of change. For example, stable-isotope analyses of carbon (δ^13^C) and nitrogen (δ^15^N) extracted from seal (pinniped) bone remains from archaeological sites in Nunatsiavut (Labrador, Canada), dating from the seventeenth to nineteenth centuries, enabled Miller *et al.* [77] to evaluate the trophic position and foraging patterns of these individuals during their lifetimes. The evidence suggests the introduction of firearms across this period had a significant effect on the size of seals targeted by hunters, with smaller and possibly younger individuals taken using traditional hunting techniques in the seventeenth century compared with later periods. Stable-isotope analysis also illuminates the effects of the introduction of industrial-scale exploitation across multiple marine mammal species from Patagonia and Antarctica [78]. In this instance, museum-stored bone samples enabled the authors to collate isotope samples from individual remains archived prior to the establishment of industrial whaling. Isotopic shifts suggest trophic and foraging changes in whales and seals across pre- and post-whaling periods. Pre-existing collections of bone were also used by Yamoah *et al.* [79] in their compound-specific isotope analyses (CSIA) of North Sea populations of Atlantic cod (*Gadus morhua*). CSIA allowed the authors to disentangle changes in the baseline of the food web from stable-isotope changes attributed to cod trophic-level changes. Against the expectation of cod trophic levels falling in response to industrialized fishing, cod populations exhibited trophic stability across the 1500-year record until a sharp increase in trophic levels in the past century. This shift was attributed to overfishing and habitat degradation producing complex ecological trophic responses or physiological changes increasing trophic enrichment factors. Collectively, these studies show the power of stable-isotope analyses of ancient biomolecules in understanding population structure. Zampirolo *et al.* [80] used a different ancient biomolecule, sequencing ancient DNA extracted from 25 sturgeon bones from the Danube River basin, to determine taxonomic identity and understand the evolutionary history of an iconic taxa. Sturgeon migrate between river and sea to complete their life cycle, making them key sentinels for human impact across ecosystems. Despite evidence of substantial exploitation, ancient DNA sequence phylogenies found limited evidence for genetic change over the Mesolithic, classical Roman and medieval periods.

Shell and bones can remain recognizable and amenable to ancient biomolecular analysis over many millennia, preserving not only data about the biological processes but also climatic changes. Schöne *et al.* [81] assessed the utility of common periwinkle (*Littorina littorea*) shells as a palaeoclimate proxy, revealing that δ^18^O values extracted from shell samples from an archaeological site in Orkney (Scotland) could accurately reconstruct past summer seawater temperatures. Data taken from the aperture of these shells revealed that periwinkles were selectively harvested during spring, a season where food sources are typically limited during the study period, suggesting they may have served as a famine food for Orkney islanders across the fourteenth to nineteenth centuries. Mollusc shells were also analysed in Reynolds *et al.* [82], where they created an annually resolved record of baseline climatic data covering more than 600 years from the North Sea, using shell growth increments from 50 individual ocean quahog (*Arctica islandica*)—the longest-lived bivalve mollusc. The resulting dataset contextualizes concurrent changes in marine ecosystems and opens the possibility of comparison with other archives of biodiversity change.

### (b) Human chronicles of the changing sea

Integrating written records, archaeological climate and palaeoecological data, articles in this section attempt to understand the complex relationship between people and changing seas. Methods include catch reconstruction, ecosystem modelling and historical ecological approaches to describe responses to cultural transitions—for example, the advent of agriculture and colonization.

Cultural shifts have been a substantial driver of biodiversity change for bivalves in the Gulf of California over hundreds of years. Walther Mendoza *et al*. [83] used archaeological records and historical documents to describe firstly how bivalves were used by Indigenous Peoples in the region, the subsequent colonial development of a large pearl fishery, and finally overharvest and functional extinction of natural oyster beds in the past century. Combining these data with stakeholder workshops revealed hopeful possibilities for restoration and aquaculture in the Gulf of California. A transdisciplinary analysis into the environmental stressors of colonization on səlilwətaɬ (Tsleil-Waututh) [84], a Coast Salish Indigenous Nation in British Columbia (Canada), illuminates the substantial social–ecological impacts of colonization. Critically, this study was conducted at the request of the səlilwətaɬ Nation in a collaborative partnership, wherein interviewees and members of the Nation were active participants in the research. An ecosystem model integrated archaeological, archival and səlilwətaɬ Nation data sources, illuminating the impacts of introduced epidemics and settler fisheries on the biodiversity and biomass in the Inlet. In a study of the broader region, using historical sources and in consultation with First Nation groups, Hayes *et al.* [13] documented a substantial shift in clam fisheries production, primarily as a result of settler actions. A stark contrast is drawn between the sustainable circular ancestral clam tending practices and commercial clam harvesting, the latter of which was responsible for biodiversity loss and in part drove the dispossession of fisheries and land from Indigenous groups.

Commercial exploitation followed colonization for many species across the North Pacific. This is shown by reconstructed Pacific cod (*Gadus macrocephalus*) catches across the Bering Sea, Aleutian Islands and Gulf of Alaska, which reveal a gradual rise and peak in the early twentieth century before a relative collapse in catches by 1950 [85]. Analysis of sea temperatures during periods of high and low catches revealed a key role of natural climatic variation, alongside fisheries, in driving decreasing cod catches. Natural climate and human activities also combined in unexpected ways across the history of the biodiverse and productive Southern Central American Isthmus. In their review of the historical marine ecology of the region, Cybulski *et al.* [86] combined historical, archaeological and palaeoecological data to reconstruct changes across five identified periods, synthesizing the ecologies of the people who inhabited the Isthmus from the Late Pleistocene to the Modern Period. The adoption of agriculture, stabilization of sea levels around 7000 BP and Spanish colonization were identified as key transitions, fundamentally altering human–ocean relationships in the region.

In the final article of this section, del Valle *et al.* [87] present a global systematic review of marine historical ecology. Through an analysis of 543 peer-reviewed articles, they provide a detailed evaluation of progress in historical marine ecology. Across all studies, the vast majority (85%) found evidence for a decline in the studied marine animal population, with increases in populations principally recovering exploited populations (e.g. whales and turtles). They also observed a bias towards commercially important taxa, with 40.7% of the articles focusing on fishes, and substantial bias towards the Global North, in both the ecosystem of focus and the affiliations of authors. This review underlines the strength of a historical ecology approach to understanding marine change and the need for wider participation and inclusion of the Global South.

### (c) Sediment records and ocean futures

In this final section, two articles reveal how ancient environmental DNA (eDNA) can be extracted from sediment records to understand past marine biodiversity. Using two sediment cores that cover over three thousand years of marine change, Holman *et al.* [88] explored the ecological effects of the human settlement of Iceland. Metabarcoding of sediment samples detected declines in the amount of eDNA from Atlantic herring (*Clupea harengus*) over the time series, which correlated with reconstructed sea surface temperatures, suggesting a role of climate in shaping herring abundance. Limited changes in the detection rate of Atlantic cod (*G. morhua*) eDNA were observed, despite contrasting evidence for substantial exploitation of this species. This underlines how sediment records might be appropriately (or inappropriately) interpreted given that they represent a localized signal. Further weaknesses and strengths of sedimentary ancient DNA in understanding marine change are outlined by Campbell *et al.* [89], who present a thorough review of the approach to understanding Australian oceans. They note the potential of the approach given the rich Indigenous history and large areas of submerged landscapes after the Last Glacial Maximum (21k years ago) and highlight a lack of sedimentary ancient DNA research to date. They identify opportunities to incorporate Indigenous knowledge with other proxies to understand the complex interplay between humans and the oceans across vast Australian seas. The issue closes with an article highlighting the enormous potential for long-term perspectives to inform marine management [90]. Although currently under-utilized, deep-time approaches such as those presented in this issue offer novel perspectives that can challenge contemporary scientific and management norms. The following three perceived barriers to the integration of long-term datasets into marine management are highlighted: a lack of data availability, a lack of data comparability and data relevance for a particular ecosystem or species. Roberts *et al.* [90] challenge each of these issues, outlining pioneering examples where deep-time data have enabled better management of the oceans.

## 4. Flourishing seas

Throughout human history, the oceans have sustained societies, inspired cultures and profoundly influenced the trajectory of civilization. Across this history, individuals have documented local ocean degradation, but only recently have we truly begun to grasp the extent of our wider impact. This issue brings together diverse disciplines and perspectives to understand deep-time sea changes. A huge range of data types, including bones, historical reports, sediment cores, documents and shells, have offered unprecedented insights into historical oceans.

The British biologist Thomas H. Huxley’s often quoted nineteenth-century belief that ‘...probably all the great sea fisheries, are inexhaustible; that is to say, that nothing we do seriously affects the number of the fish’ contrasts with the first emergent theme from this issue—that the oceans are in constant change, and that no sea is truly inexhaustible. Contributions in this issue have shown natural shifts in animal populations across millennia, for example, Atlantic herring (*C. harengus*) responding to sea temperature shifts long before human settlement in Iceland [88], or sea level changes reshaping coastlines and ecosystems in Australia [89] and Central America [86]. Moreover, alongside the rhythmic waxing and waning of ocean conditions, this issue has documented continuous cultural and technological changes within human societies linked with the marine world. For example, maritime technologies enabled long-distance ocean travel, allowing seafarers to target remote species such as marine mammal species in the Southern Ocean [78] and bringing previously isolated cultures into contact [84,86]. Other technologies allowed people to change their access to food, such as the development of firearms enabling North American Indigenous hunters to target different seals in a population [77], or the broad-scale industrialization of fisheries in North America [13,85] and Europe [79]. Each of these cultural or technological shifts had an impact on local marine ecosystems. Whether subtle or substantial, our cultural revolutions have cast long shadows across the seas. Collectively, this evidence underlines the importance of considering climate, human culture and marine ecosystems holistically, particularly as changes in the systems have often occurred simultaneously [85,86,88]. To truly understand changing oceans, a full accounting of all possible drivers—anthropogenic and natural—may be required.

Capturing this complexity is itself a complex task, and a second key theme from these contributions is how the application of novel techniques in interdisciplinary research has enabled new or unique findings. In this issue, contributions have shown how we can now reconstruct past ocean conditions—for example, temperature [81] or productivity [82]—allowing us to turn back the clock and learn about the climate of ancient oceans. Other molecular techniques have documented past ecosystems in great detail, for example, by showing biodiversity changes [80,88,89] or through reconstructing changes in food web structure [78,79]. Novel methods like CSIA [79] and sequencing of ancient eDNA [88,89] allow us to understand the state of ocean ecosystems long before monitoring programmes began, and thus hold great promise in challenging existing paradigms and resolving intractable issues in ocean history. Beyond these important technological developments, the integration of methods, new and old, in interdisciplinary studies has revealed key insights into the oceans’ past. For example, by combining catch reconstructions with sea surface temperature data to appreciate how fishing and climate comparatively contributed to Pacific cod decline, as shown in McClenachen *et al.* [85], or through combining Indigenous knowledge with numerical ecosystem modelling to develop a more holistic and complete understanding of the effect of colonization on the Burrard Inlet, as shown in Efford *et al.* [84]. These studies remind us that both interdisciplinary research and novel methods can uncover findings that might elude investigation within a single discipline.

A third key emergent theme is the pervasive effect of colonization on human–ocean interactions, with contributions from both the Atlantic [77] and Pacific [13,83,84,86] coasts of the Americas, as well as Australia [89], demonstrating how European colonization disrupted local Indigenous communities and marine ecosystems. The collapse of both shellfish [13,83,84] and finfish [84,85] populations after the colonization of the Pacific coast of North America serve as examples of how previously thriving populations under Indigenous management were quickly exhausted under the combined strain of industrial fishing and Indigenous dispossession. While there remain substantial research gaps in the Global South, as shown by del Valle *et al.* [87], these studies agree with previous research revealing a shift towards unsustainable industrial exploitation of marine resources and dispossession of Indigenous Peoples [10,35]. However, just as reconstructing past marine animal abundance offers hopeful possibilities for the future, the human–ocean relationships developed by Indigenous Peoples are not lost and can contribute to marine management in the future.

So, what do the articles in this issue suggest for future seas? First, they lay out a clear direction of travel for future research. As outlined above, interdisciplinarity and the application of novel methods will continue to yield unique and unexpected findings. However, this approach is likely to make the largest impact across currently unstudied regions—for example, Africa and Asia, where exciting ocean histories lie beneath the waves. Will these regions, with their unique human cultures and marine biodiversity, show similar changes to well-studied seas? Or will they provide new examples of seas shifting in hitherto unseen ways? Future research may also benefit from seeing people as part of, not separate from, biodiversity and climate. Can we, for example, better understand changing fisheries by modelling humans as both consumers and a symbiotic part of ocean biodiversity, informed by the many examples [75] of Indigenous Peoples enhancing marine ecosystems?

Beyond the world of research, the contributions in this issue may have wider implications for ocean futures. We live in a period of challenge and rapid environmental change as the intertwined biodiversity and climate crises reshape seas globally. However, the ability of marine ecosystems to recover from impact should fill us with hope and galvanize us into action. Despite continued exploitation, some marine animal populations have shown recovery in the past 50 years [91,92]. Datasets like those presented in this issue provide marine managers with baseline data against which to compare their efforts to restore ocean ecosystems. This is critical, as the number of restoration initiatives appears to be rapidly increasing across the globe [91], while observational datasets from which an ecosystem baseline might be drawn rarely exceed a century [93]. Insights from past oceans offer not only ecological baselines but also lessons about the human-driven processes through which degradation occurs [13,84,85]. As the conditions of our planet change, we can turn once again to information from oceans past, allowing us to revisit or learn from past ways of sustainable interaction in the present. Some examples might include sustainable gardening of coastal bivalves as found in the Pacific Northwest of North America [13], maintaining natural resources well above the current level of need as found in some Aboriginal Australian contexts [89,94], or exercising caution with new technologies that have unforeseen ecosystem-wide impacts, as seen by the industrialization of fishing [79] or the introduction of firearms [77]. Just as diverse marine communities thrive in an ever-changing ocean, diversity in human–ocean interactions will help future societies succeed in the face of a changing Earth.

## Data Availability

This article has no additional data.
